# Bumble bee gut microbial community structure differs between species and commercial suppliers, but metabolic potential remains largely consistent

**DOI:** 10.1128/aem.02036-24

**Published:** 2025-02-06

**Authors:** Michelle Z. Hotchkiss, Alexandre J. Poulain, Jessica R. K. Forrest

**Affiliations:** 1 Department of Biology, University of Ottawa 151176 , Ottawa, Ontario, Canada; Norwegian University of Life Sciences, Ås, Norway

**Keywords:** bumble bees, *Bombus*, gut microbiotas, metagenomics, wild bees, commercial bees, metabolic potential

## Abstract

Bumble bees are key pollinators for natural and agricultural plant communities. Their health and performance are supported by a core gut microbiota composed of a few bacterial taxa. However, the taxonomic composition and community structure of bumble bee gut microbiotas can vary with bee species, environment, and origin (i.e., whether colonies come from the wild or a commercial rearing facility), and it is unclear whether metabolic capabilities therefore vary as well. Here we used metagenomic sequencing to examine gut microbiota community composition, structure, and metabolic potential across bumble bees from two different commercial *Bombus impatiens* suppliers, wild *B. impatiens*, and three other wild bumble bee species sampled from sites within the native range of all four species. We found that the community structure of gut microbiotas varied between bumble bee species, between populations from different origins within species, and between commercial suppliers. Notably, we found that *Apibacter* is consistently present in some wild bumble bee species—suggesting it may be a previously unrecognized core phylotype of bumble bees—and that commercial *B. impatiens* colonies can lack core phylotypes consistently found in wild populations. However, despite variation in community structure, the high-level metabolic potential of gut microbiotas was largely consistent across all hosts, including for metabolic capabilities related to host performance, though metabolic activity remains to be investigated.

**IMPORTANCE:**

Our study is the first to compare genome-level taxonomic structure and metabolic potential of whole bumble bee gut microbiotas between commercial suppliers and between commercial and wild populations. In addition, we profiled the full gut microbiotas of three wild bumble bee species for the first time. Overall, our results provide new insight into bumble bee gut microbiota community structure and function and will help researchers evaluate how well studies conducted in one bumble bee population will translate to other populations and species. Research on taxonomic and metabolic variation in bumble bee gut microbiotas across species and origins is of increasing relevance as we continue to discover new ways that social bee gut microbiotas influence host health, and as some bumble bee species decline in range and abundance.

## INTRODUCTION

Bumble bees (*Bombus* spp.; Hymenoptera: Apidae) are eusocial bees that are key pollinators in many ecosystems. Bumble bees not only pollinate many wild plants (1) but also crops (2–8), particularly those that require buzz pollination, a service that honey bees cannot perform. Understanding factors that influence bumble bee health is therefore important to maintain the viability and productivity of plant populations and communities, especially as some bumble bee species are experiencing considerable reductions in range and abundance (9, 10). One important facet of bumble bee health that has gained attention in recent years is their gut microbiota (11).

Social, corbiculate bees, a group that includes honey bees, bumble bees, and stingless bees, all have a highly conserved, core gut microbiota (11–13). This microbial community primarily supports host health by inhibiting pathogen infection (14–19) and stimulating the host immune system (20–23), though a suite of other benefits has also been observed (24–32). Typically, social bee gut microbiotas are dominated by five core phylotypes (a core phylotype being a microbial taxon that is consistently present within a given host taxon) representing five bacterial taxa: *Bifidobacterium* spp. (*Bifidobacteriaceae*), *Bombilactobacillus* spp. (*Lactobacillaceae*), *Gilliamella* spp. (*Orbaceae*), *Lactobacillus* spp. near *melliventris* (*Lactobacillaceae*), and *Snodgrassella* spp. (*Neisseriaceae*) (11, 13). However, taxonomic composition can differ across host species and environments, particularly in stingless bees and bumble bees (12, 33).

Bumble bee gut microbiotas have been profiled across a variety of species and environments (e.g., 12, 14, 34–40), and patterns in community composition and structure have begun to emerge. Core social bee gut microbial phylotypes generally dominate bumble bee gut microbiotas, with *Gilliamella* and *Snodgrassella* being the most consistently present (e.g., 11, 34, 35, 37, 38, 41–44). However, the presence of some core phylotypes, particularly *Bombilactobacillus* and *Lactobacillus,* varies between host species (12, 37). Furthermore, some *Bombus* species have gained the additional core phylotypes *Candidatus* Schmidhempelia (hereafter *Schmidhempelia*) (*Orbaceae*), and/or *Bombiscardovia* (*Bifidobacteriaceae*) (12, 37, 38, 45, 46). Regardless of species, wild bumble bees, which forage in outdoor environments, have more non-core phylotypes (i.e., microbial taxa that are sporadically present in a given host taxon) in their gut microbiotas than laboratory-reared bees. These non-core phylotypes include other corbiculate bee-associated microbes such as *Apibacter* (37, 43, 47), and environmental microbes, like *Fructobacillus,* other non-core *Lactobacillaceae*, and *Enterobacteriaceae* (34, 35, 37, 38, 41–44, 47). In some cases, these non-core phylotypes can displace core phylotypes and become dominant gut microbiota constituents (35, 38), as is the case in many bumble bees collected from forest environments (34, 42). Meanwhile, laboratory-reared bees who are prevented from foraging outdoors tend to have few, if any, non-core phylotypes present; if present, non-core phylotypes tend to occur at low abundances (11, 48–52).

In addition to species and rearing environment, whether bumble bees come from a commercial supplier or a wild population (i.e., bee origin) can also influence their gut microbiota taxonomic composition. Companies began to rear bumble bee colonies commercially in the late 1980s for agricultural pollination in fields and greenhouses (53). Commercial stocks also present a quick and easy way for researchers to obtain fully developed bumble bee colonies for experiments at any time of year, and lab studies on bumble bee gut microbiotas using commercial bees are becoming increasingly common (e.g., 31, 48, 49, 54–57). These stocks, which mainly comprise two species (the North American *B. impatiens* and the European *B. terrestris*), represent populations that have been reared in indoor facilities for generations with no access to the outside environment. Generally, commercial colonies harbor the same core phylotypes as wild bees (e.g., 31, 49, 54, 58). However, recent studies have observed that commercial *B. impatiens* colonies from some suppliers can lack *Gilliamella,* a core phylotype that is consistently present in wild *B. impatiens* populations (48, 49).

The observation that gut microbiota community composition varies with bumble bee species, environment, and origin raises the question: Does this variation in taxonomic composition beget variation in gut microbial community function? The answer is unclear. Extensive work has been conducted to describe the metabolic capabilities of core social bee gut microbes. Multiple core microbial strains have been isolated from social bee species, including bumble bees, and had their genomes fully sequenced (e.g., 28, 30, 46, 59–68), providing extensive information on gene content and metabolic potential. *In vitro* growth experiments (e.g., 15, 24, 28, 30, 69) and *in vivo* colonization experiments with these strains (e.g., 24, 26, 70) have experimentally validated the inferred metabolic functions. Based on this work, we know that *Bombilactobacillus*, *Bombiscardovia*, *Bifidobacterium*, *Gilliamella*, *Schmidhempelia,* and *Lactobacillus* all primarily ferment sugars, while *Snodgrassella* fuels its metabolism with fermentation products (i.e., organic acids) (13, 70). Many non-core taxa present in *Bombus* gut microbiotas also ferment sugars (71, 72). This functional redundancy present across bumble bee gut microbes may indicate that despite variation in taxonomic composition, metabolic potential remains somewhat consistent across *Bombus* populations, as it is between *B. terrestris* and honey bees (73). However, among social bee core phylotypes, phylotype-specific metabolic pathways still exist—for example, a complete citric acid cycle is present in *Snodgrassella* but lacking in all other core phylotypes (24, 26, 28). There is also additional intra-phylotype variation in metabolic capabilities (e.g., 61, 67, 69). Consequently, social bee gut microbiotas with similar phylotype composition can nevertheless differ in metabolic potential (59).

Most bumble bee gut microbiota community profiling to date has used 16S rRNA gene amplicon sequencing, a technique that provides little information on intra-phylotype diversity and no information on functional gene content (74, 75). Thus, we know little about the sub-phylotype-level composition and metabolic potential of whole bumble bee gut microbiotas. If the phylotype-level taxonomic differences we observe between bumble bee gut microbiotas from different host species and populations correspond to differences in community function, then research conducted on a single host population could be of limited relevance to other populations. For example, understanding the degree to which community function in commercial bumble bee gut microbiotas reflects that in wild gut microbiotas, and determining whether it differs between commercial suppliers, will be useful for designing experiments and interpreting results.

In this study, we use shotgun metagenomics to determine how genome-level taxonomic composition and metabolic potential in bumble bee gut microbiotas vary with host species and origin. Specifically, we examine the gut microbiotas of commercial *B. impatiens* from two different commercial suppliers, wild *B. impatiens*, and three other wild bumble bee species, *B. rufocinctus*, *B. ternarius,* and *B. vagans*, whose gut microbiotas have not been profiled before. These bumble bee populations sit at either end of the environment–origin spectrum: commercial bumble bees have been reared indoors and have never foraged outside, while wild bees come from colonies established by outdoor-foraging queens where there is abundant movement of workers between the hive and the outdoor environment. To our knowledge, this study is the first to compare genome-level taxonomic composition and metabolic potential of whole bumble bee gut microbial communities between commercial bumble bee colonies from different suppliers and between commercial and wild bumble bee populations.

## MATERIALS AND METHODS

### Bumble bee gut microbiota sampling

**Fig 1:**
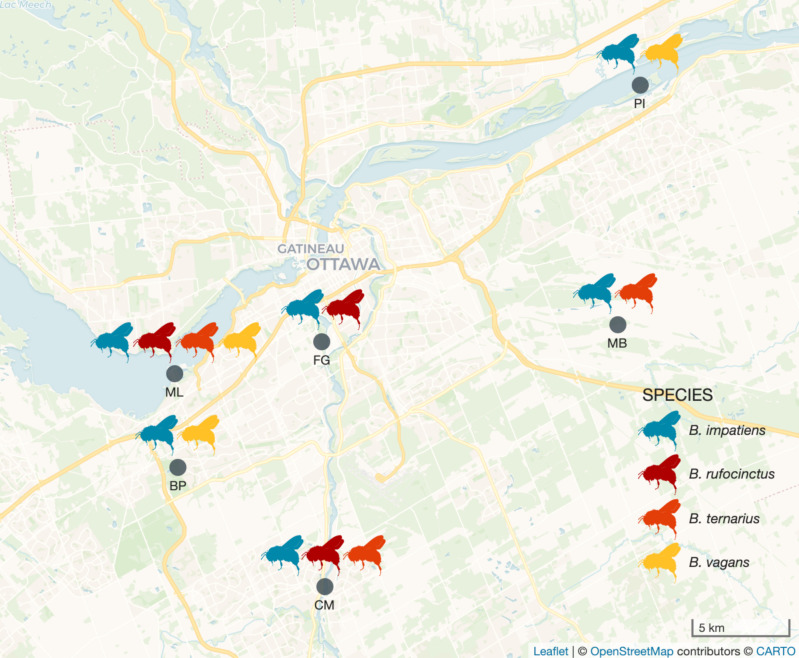
Map of wild bumble bee collection sites and species collected. Gray dots represent the locations of field sites (BP: Bruce Pit, CM: Chapman Mills Conservation Area, FG: Fletcher Wildlife Garden, MB: Mer Bleue Bog, ML: Mud Lake, PI: Petrie Island). Bumble bee icons above each site represent the species from that site from which we sampled gut microbiotas.

In this study, we investigated the gut microbiotas of both commercial and wild bumble bees, all representing species native to our study area in eastern North America. In August 2023, we purchased three *Bombus impatiens* colonies from Biobest (Leamington, Ontario, Canada) and three *B. impatiens* colonies from Koppert (Scarborough, Ontario, Canada). Upon colony arrival, we removed two workers per colony and euthanized them by freezing at −20°C. During the same month, we also collected wild bumble bees from six sites in Ottawa, Ontario, Canada: Bruce Pit, Chapman Mills Conservation Area, Fletcher Wildlife Garden, Mer Bleue Bog, Mud Lake, and Petrie Island (Fig. 1); all sites were at least 4 km apart. We opportunistically caught bumble bees at our sites using 50 mL Falcon tubes and identified them as morphospecies in the tubes. If we identified a bumble bee as a worker of an at-risk species (*B. bohemicus*, *B. pennsylvanicus*, or *B. terricola*), a queen, or a male, it was released. If not, we moved the tube containing the bee to a cooler filled with ice packs. At the end of each day, we returned to the lab and moved the bees from the cooler to a −20°C freezer to be euthanized. We assigned all bees collected in this study a unique identifier (“bee ID”) which included its species, site or colony, and replicate number. When sampling, we were unable to control for the age of sampled bees, a factor that can influence bumble bee gut microbiota taxonomic composition and structure (52). However, we do not believe variation in age greatly impacted our results (see Discussion).

Once the sampled bees were dead, we removed them briefly from the freezer to identify them to species using the dichotomous keys and images in Williams et al. (2014) (76). Ultimately, due to the number of individuals we had collected and their distribution across sites, we decided to investigate the gut microbiotas of four of the wild species we caught: the common eastern bumble bee (*B. (Pyrobombus) impatiens*), the red-belted bumble bee (*B. (Cullumanobombus) rufocinctus*), the tri-colored bumble bee (*B. (Pyrobombus) ternarius*), and the half-black bumble bee (*B. (Pyrobombus) vagans*). We collected *Bombus impatiens* from each of our six sites and collected the other bee species from three sites each; we collected all four species at Mud Lake (Fig. 1).

In this study, we refer to Biobest *B. impatiens*, Koppert *B. impatiens*, wild *B. impatiens,* wild *B. rufocinctus,* wild *B. ternarius*, and wild *B. vagans* collectively as “bee hosts.”

### Gut dissections and enrichment for prokaryotic cells

We dissected the whole guts (i.e., crop, midgut, and hindgut) of two bumble bee workers per bee host per site/commercial colony. Thus, we sampled six guts per host except for wild *B. impatiens*, for which we sampled twelve guts; four guts per site except at Chapman Mills Conservation Area and Mud Lake, at which we sampled six and eight guts respectively; and two guts per commercial colony.

We carried out all dissections in a sterile laminar flow hood. We performed the dissections in sterile tissue culture dishes, using a new dish for each bee and sterilizing all dissection tools with 10% bleach and 70% ethanol between each dissection. We did not surface sterilize bees (77).

After gut dissections and prior to DNA extraction, we enriched our samples for prokaryotic cells relative to eukaryotic (i.e., host or pollen) cells using a protocol adapted from Ellegaard and Engel (2019) (60). We placed each dissected gut in a sterile 1.5 mL Eppendorf tube filled with 250 µL of sterile 1 mm glass beads and 500 µL of sterile 1M phosphate-buffered saline (PBS). We then homogenized guts with an Omni Bead Ruptor 4 Homogenizer (Gerogia, USA) for 30 seconds at speed 4, centrifuged samples at 2,500 rpm for 5 minutes, and collected the supernatant in new, sterile tubes. Next, we centrifuged samples at 9,000 rpm for 15 minutes, discarded the supernatant, and resuspended pellets in 800 µL of 1M PBS. We then centrifuged samples for a second time at 2,500 rpm for 5 minutes. After this step, we passed the supernatant through 10 µM pluriStrainer Mini filters (California, USA) by centrifuging samples at 9,000 rpm for 30 seconds. We finished by centrifuging at 10,000 rpm for 15 minutes, removing the supernatant, and resuspending the pellets in 750 µL of the bead-beating solution from the QIAGEN DNeasy PowerLyzer PowerSoil Kit (Hilden, Germany). Each 750 µL volume was then moved to a bead-beating tube from the QIAGEN kit to begin DNA extraction.

### DNA extractions and sequencing

We extracted DNA from our samples using the QIAGEN DNeasy PowerLyzer PowerSoil Kit (Hilden, Germany), following protocol with the following modifications: (1) after adding 60 µL of solution C1 and vortexing samples, we incubated samples in a water bath for 10 minutes at 65°C, (2) we conducted all centrifugation steps at 13,000 × *g*, and (3) we allowed solution C6 to sit on the membrane for 5 minutes before elution. We quantified DNA using the Qubit 2.0 and the Invitrogen Qubit dsDNA HS Assay Kit, following protocol with the modification that the DNA was incubated in the dye/buffer mixture for 5 minutes. We sent extracted DNA to Genome Quebec for library preparation and metagenomic shotgun sequencing using the NovaSeq 6000 system; two *B. ternarius* samples, one *B. vagans* sample, and a negative DNA extraction control failed at the library creation step and so were excluded from further analysis.

### qPCR for 16S rRNA gene copy number

We used qPCR to obtain 16S rRNA gene copy numbers, a proxy for microbial abundance, from all gut samples. We generated a four-step standard curve of known 16S rRNA gene copy numbers using genomic DNA (78, 79) extracted from NEB 5-alpha competent *Escherichia coli* K-12 which we ran in triplicate on every plate; the steps of this curve were 600, 6000, 60,000, and 600,000 copies/µL. We diluted gut DNA extracts 1:150 in nuclease-free water and ran all samples in triplicate following the protocol in Motta et al. (80) and using the same universal bacterial primers (27F/355R). We used BioRad SsoFast EvaGreen Supermix with a BioRad CFX96 real-time system and C1000 thermocycler, and analyzed run data with BioRad CFX Maestro software (v.2.3). We verified melt curves for each run and accepted run efficiencies of 85%–110%.

### Metagenomic bioinformatics

We had an average of 66 million paired-end raw reads per sample (range: 51.4 million–79.9 million). We examined the quality of raw reads using FastQC v0.11.9 (81), and trimmed and filtered out low-quality reads using fastp v0.23.2 with default settings (82). After this filtering step, we had an average of 65.6 million paired-end reads per sample (range: 50.9 million – 79.3 million). We then used Bowtie 2 v2.5.1 (83) with the --very-sensitive flag to map reads against host genomes and retain unmapped reads. For *B. impatiens* samples, we mapped reads against a *B. impatiens* genome (NCBI GCF_000188095.3) (84). However, *B. rufocinctus*, *B. ternarius*, and *B. vagans* genomes have not been sequenced, so we used a published phylogeny to determine their closest relative with a sequenced genome (85). Consequently, we mapped *B. rufocinctus* reads against a *B. cullumanus* genome (GenBank GCA_014737535.1), *B. ternarius* reads against a *B. bifarius* genome (NCBI GCF_011952205.1), and *B. vagans* against a *B. impatiens* genome (NCBI GCF_000188095.3) (64, 84, 86). An average of 68% of paired reads per sample mapped to the host or host-related genome (range: 9.2%–96.4%); after this filtering step, we were left with an average of 20.4 million paired-end reads per sample (range: 2.3 million–57.2 million) (Fig. S1, S2A and B).

We assembled filtered reads for each sample using MEGAHIT v1.2.9 with the --no-mercy flag (87). We assessed assembly quality using quast v5.0.2 and multiqc v1.9 (88, 89). We binned contigs from each sample into metagenome-assembled genomes (MAGs) using CONCOCT v1.1.0 with a minimum contig size of 1,000 bp, MetaBAT 2 v2.15 with a minimum contig size of 1,500 bp, and Maxbin 2.0 v2.2.7 with a minimum contig size of 500 bp. We refined MAGs within each sample using DAS Tool v1.1.6 (90). We assessed the quality of all MAGs using CheckM2 v1.0.1 (91) and assigned taxonomy using GTDB-Tk and the Genome Taxonomy Database (GTDB) (92, 93).

Due to low-read counts after host-filtering, some bumble gut microbiota samples yielded few or no high-quality MAGs. Therefore, to determine the taxonomic structure of each sample, we decided to map reads from each sample against a pooled set of high-quality MAGs from across all samples. Across all our bee gut microbiota samples, many MAGs were assigned to identical taxonomic groups and therefore presumably had high shared average nucleotide identities (ANIs). When mapping sample reads against a set of MAGs to determine the relative abundance of each MAG in the sample, the presence of many MAGs with high shared ANIs (i.e., >98%) can present issues for mapping tools (94). Therefore, we dereplicated MAGs using drep with a secondary ANI threshold of 98% to obtain a set of representative MAGs for mapping (95). After dereplication, we were left with 33 MAGs, all of which had >80% completeness and 31 of which had <10% contamination (i.e., redundancy).

Both MAGs that had >10% contamination were assigned to *Fructobacillus tropaeola* and were the only MAGs for this taxon in our dereplicated set. Thus, we attempted to manually refine these MAGs so that this taxon would not be removed entirely. We used MMseqs2 to assign taxonomy to all contigs in each contaminated MAG using the GTDB and then removed any contigs that had been assigned to genera other than *Fructobacillus* (93, 96). This technique decreased the contamination of one *F. tropaeola* MAG below 10% (from 10.14% to 7.75%), so we retained the refined version of the MAG in our representative set; we discarded the other MAG because contamination remained high (19.62%) even after manual refinement.

Thus, after dereplication and manual refinement, we had a set of 32 dereplicated MAGs, all with >80% completeness (median = 99.5%) and <10% contamination (median = 0.3%) (Table S1). For each MAG, we created a taxonomic ID consisting of the lowest taxonomic assignment for that MAG (92) and, when necessary, a number to distinguish between congeneric and conspecific MAGs. We used GToTree v1.8.4 to infer an approximate maximum-likelihood phylogenetic tree of the representative MAGs using the program’s “Bacteria” set of 74 bacterial single-copy genes (97).

We used CoverM v0.6.1 with a minimum percent identity of 95% and a minimum read aligned percent of 75% to map host-filtered reads from each bee gut microbiota against the set of representative MAGs to determine the coverage and relative abundance (i.e., relative reads per kilobase per million mapped reads) of each MAG in each bee gut microbiota (98). The mean percentage of unmapped reads was 42.5% (range: 3.1%–96.7%), with *B. vagans* having the highest average unmapped percentage across all hosts (Fig. S2E). To investigate the reason for this high percentage of unmapped reads, we mapped host-filtered reads for each *B. vagans* sample against the Kraken 2 nt database, which contains both eukaryotic and prokaryotic sequences, to determine which non-microbial taxa were present (99).

After determining the MAG-inferred taxonomic community structure of our bee gut microbiotas, we wanted to examine whether gut microbiota metabolic potential varied with bee host and, for wild bees, by site. To accomplish this, we first had to determine the metabolic potential of each representative MAG. However, if we only examined the metabolic functions present in our representative MAGs, we could miss accessory functions found in closely related MAGs (i.e., shared ANIs > 98%) that were removed during dereplication (94). To solve this issue, we took all MAGs refined by DAS Tool that were removed during dereplication and selected those with >80% completeness and <10% contamination. We then used shared ANI phylogenies produced by drep to assign each high-quality, DAS Tool-refined MAG to the representative MAG with which it shared the highest ANI (95); to avoid pseudoreplication, if two MAGs were from the same bee host and site/colony, had a shared ANI > 99.5%, and were assigned to the same representative MAG, only the highest-quality MAG of the pair was retained for analysis. We called the resulting groups, which each consisted of a representative MAG and any related high-quality DAS Tool-refined MAGs, MAG clusters. We assigned these MAG cluster IDs identical to the taxonomic ID of the cluster’s representative MAG. This clustering process resulted in 71 MAGs being assigned to MAG clusters for a total of 103 MAGs for subsequent metabolic analyses (71 high-quality DAS Tool MAGs + 32 representative MAGs); these MAGs had a median completeness of 95% and a median contamination of 0.37% (Table S2). To visualize relatedness between MAG clusters, we used GToTree v1.8.4 to infer an approximate maximum-likelihood phylogenetic tree using 74 bacterial single-copy genes (97). We then annotated metabolic functions in all 103 clustered MAGs using the KEGG module database and Anvi’o, specifically the anvi-gen-contigs-database, anvi-run-kegg-kofams, and anvi-estimate-metabolism programs with the stepwise module completion threshold set at 0.75 (100–102).

After annotating the KEGG modules in each MAG cluster, the next step was to determine whether the same KEGG modules were present across all bee gut microbiotas regardless of MAG cluster (i.e., taxonomic) composition. Ideally, we would have simply annotated the functions of each sample’s set of MAGs. However, some samples contained no high-quality MAGs, and MAGs had to be pooled across samples for taxonomic profiling (as described above), so we had to use a more complex approach. First, we calculated the relative presence of each KEGG module in each MAG cluster (i.e., the number of MAGs in a MAG cluster with a given module/the total number of MAGs in that cluster). Second, we cataloged all KEGG modules that could be present in each bee gut microbiota based on which MAG clusters were present (i.e., which representative MAGs reads mapped to during coverage mapping); we considered a MAG cluster present in a bee gut microbiota if its representative MAG had a relative abundance >1%. Third, we calculated the minimum probability that each KEGG module was actually present in a given bee gut microbiota based on the relative presence of each metabolic pathway in all MAG clusters present in that gut microbiota. As a hypothetical example, if a gut microbiota contained the *Apibacter 1* and *Bifidobacterium 1* clusters, the relative presence of module A was 0.5 in *Apibacter 1* and 0.75 in *Bifidobacterium 1*, and we know that the gut microbiota must contain at minimum one MAG per MAG cluster, then the minimum probability that module A is present in the gut microbiota would be 1 − (probability that neither the *Apibacter 1* nor *Bifidobacterium 1* MAG contains module A), or 1 – ((1–0.5)*(1–0.75)), or 0.875. We performed this calculation for each KEGG module that could be present in each bee’s gut microbiota. Based on this information, for each module, we also calculated the percent of individuals for each bee host whose gut microbiotas contained that module (i.e., the number of gut microbiotas for each bee host in which the minimum probability of module presence ≥0.75/the total number of gut microbiotas sampled for that host).

### Statistical analyses

We conducted all statistical analyses in R v4.3.1 (103). Detailed descriptions of the univariate and multivariate analyses we ran are described in subsequent paragraphs and code is available (see Data availability). The overarching objective of our analyses was to determine how various metrics (e.g., microbial abundance, community composition, etc., see below) differed between bee hosts (i.e., between Biobest *B. impatiens*, Koppert *B. impatiens*, wild *B. impatiens,* wild *B. rufocinctus,* wild *B. ternarius*, and wild *B. vagans*) and, for wild bees, between sites. Therefore, for each metric we typically ran two models: one using data from all bees with the metric of interest as the dependent variable and bee host as an independent variable with six levels (“host model”), and another using data from only wild bees, with the metric of interest as the dependent variable and both site (six levels) and host (four levels) as independent variables (“site model”). The inclusion of bee hosts in the site model was to control for variation among hosts when testing for differences across sites; it had insufficient levels to be coded as random and was therefore coded as fixed. As differences between bee hosts were already tested in the species models, results for differences between species in site models were not reported.

Across all analyses, we fit linear and generalized linear models using the “lm,” “glm,” and “glm.nb” functions in the MASS and base R stats packages (103, 104). Often for linear models, the assumptions of normality and homoscedasticity were violated; in these cases, we used non-parametric tests with ranked dependent variables. We determined the significance of terms in linear and generalized linear models using Type 3 analyses of variance (ANOVAs) with the “Anova” function from the car package and examined model assumptions using the performance package (105, 106). We performed post hoc tests using the “TukeyHSD” function in the stats package, the “dunnTest” function in the FSD package, and the emmeans package, all with Bonferroni corrections (103, 107, 108). We manipulated and visualized all data using the R packages dplyr, ggplot2, ggtree, patchwork, and leaflet (109–114).

We analyzed variation in host DNA filtering, microbial abundance, and representative MAG set mapping using linear host and site models with log_10_ number of reads post-host filtering, log_10_ 16S rRNA gene copy count, and percent of reads unmapped as the dependent variables, respectively. All initial linear models showed signs of heteroscedasticity, so we reran models with ranked dependent variables and conducted post hoc analyses using Dunn’s test (108).

We conducted taxonomic analyses at two resolutions: phylotype (i.e., genus) and representative MAG. We used the vegan package to determine phylotype and representative MAG richness and Shannon diversity for each bee gut microbiota (115). We employed generalized linear models with Poisson distributions to analyze variation in phylotype and representative MAG richness with respect to bee host and site; the model analyzing MAG richness with respect to bee host was overdispersed, so we reran the model with a negative binomial distribution. Post hoc tests for richness models were conducted with the emmeans package (107). We used linear models with ranked dependent variables to analyze variation in phylotype and representative MAG Shannon indices as functions of bee host and site.

To analyze how phylotype and representative MAG community structure in bee gut microbiotas varied with bee host and site, we used the vegan and ape packages in R to generate Bray-Curtis dissimilarity matrices of bee gut microbiota community structure (115, 116). We then used the “adonis2” function in the vegan package to conduct permutational analyses of variance (PERMANOVAs) with 9,999 permutations (115). We evaluated the homogeneity of group dispersions using the “betadisper” function in the vegan package with the type set to “median” (115). We conducted post hoc comparisons using the pairwiseAdonis package for PERMANOVA models and the “TukeyHSD” function for differences in group dispersions (117). We also visualized differences using principal coordinates analyses (PCoAs). To identify which phylotypes were differentially abundant across bee hosts, we used a series of linear models with ranked dependent variables. For each model, the relative abundance of a given phylotype was the dependent variable and bee host was the independent variable. If the species term in a model was significant based on a Type 3 ANOVA, we conducted a post hoc analysis, using Dunn’s test to determine which bee species were significantly different (108). As we had over 30 representative MAGs and many of their relative abundances clearly varied with bee host (i.e., most MAGs were completely absent from at least one host; see Results), we elected not to formally analyze which representative MAGs were differentially abundant across bee hosts.

To visualize differences in the metabolic potential of MAG clusters, we used the vegan and ape packages in R to generate a Jaccard distance matrix of KEGG module presence and absence in MAG clusters (115, 116), which we then used for PCoA. To examine whether any KEGG modules were differentially enriched across MAG clusters (i.e., if any modules were consistently present in some MAG clusters but consistently absent in others), we used the Anvi’o program anvi-compute-metabolic-enrichment with the stepwise module completion threshold set to 0.75 (100, 118). This analysis provides two values: an unadjusted *P*-value, and a q-value adjusted for false detection rates. In our case, adjusted q-values were lower than unadjusted *P*-values; to be conservative, we report results using unadjusted *P*-values.

Finally, to investigate whether the metabolic potential of bee gut microbiotas varied with bee host or, for wild bees, by site, we used the vegan and ape packages in R to generate Jaccard distance matrices of KEGG module presence and absence in the gut microbiotas of all bees and wild bees only (115, 116); a KEGG module was considered “present” in a bee gut microbiota if its minimum probability of presence was ≥0.75. We visualized these data using PCoAs. We then used the “adonis2” function in the vegan package to conduct permutational analyses of variance (PERMANOVAs) with 9,999 permutations to analyze how the metabolic potential of bee gut microbiotas varied with bee host and, for wild bees, with the site (115). We evaluated the homogeneity of group dispersions and conducted post hoc comparisons as described above for community structure. To identify which KEGG modules were differentially enriched across bee hosts, we manually compiled a file with the same structure as the “modules” mode output file from the Anvi’o program anvi-estimate-metabolism (102). In this file, we listed a module as being present in a bee gut microbiota (i.e., stepwise_module_is_complete = TRUE) if its minimum probability of presence was ≥0.75. We then used that file with the Anvi’o program anvi-compute-metabolic-enrichment (118) to calculate enrichment scores, unadjusted *P*-values, and adjusted q-values. In this case, the adjusted q-values were higher than the *P*-values, and so we used q-values to determine module enrichment significance.

## RESULTS

### Read mapping and 16S rRNA gene copy counts

The number of reads that remained after mapping to host or host-related genomes did not vary with bee host (*F*_5,33_ = 1.35, *P* = 0.27) or with site for wild bees (*F*_5,18_ = 1.24, *P* = 0.33) (Fig. S1 and S2), though the average number of reads post-host filtering for *B. ternarius* was an order of magnitude lower than that for all other hosts (10^6^ vs. 10^7^).

Bumble bee gut microbiota 16S rRNA gene copy counts, a proxy for microbial abundance, varied with bee host (*F*_5,33_ = 4.00, *P* = 0.006) but not site (*F*_5,18_ = 2.14, *P* = 0.11) (Fig. S2). Specifically, gene copy counts were significantly lower in *B. vagans* gut microbiotas than in those of Biobest *B. impatiens* (|Z| = 3.06, *P =* 0.03) (Fig. S2A).

After mapping reads against the set of representative MAGs, the percentage of unmapped reads varied significantly with bee host (*F*_5,33_ = 11.5, *P* < 0.0001) but not site (*F*_5,18_ = 2.65, *P* = 0.06) (Fig. S2). The percentage of unmapped reads was higher in *B. vagans* gut microbiotas than in Koppert *B. impatiens* or wild *B. impatiens* microbiotas (all |Z| > 3.69, all *P <* 0.01). Subsequent mapping of host-filtered reads from *B. vagans* microbiotas against a database containing eukaryotic and prokaryotic sequences revealed an average of 91.3% of reads mapped to the genus *Bombus*.

### Taxonomic community structure

**Fig 2:**
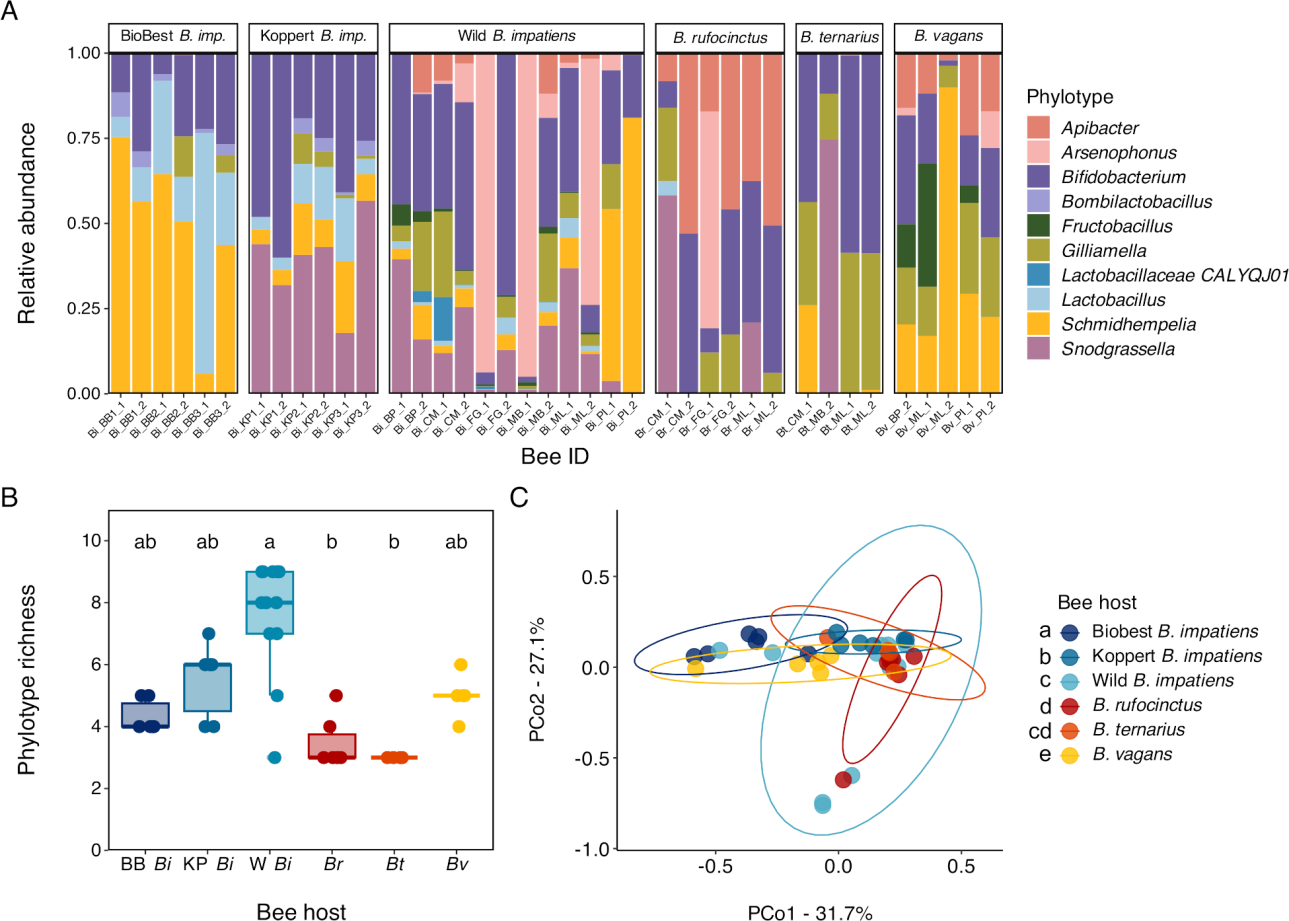
Bee gut microbiota diversity at the phylotype level. (**A**) Stacked bar plot of relative abundances of microbial phylotypes in gut microbiotas faceted by bee host. (**B**) Box plot of phylotype richness by bee host. Boxes represent medians and interquartile ranges; whiskers extend to 1.5 × the interquartile range. BB *Bi* = Biobest *B. impatiens*, KP *Bi* = Koppert *B. impatiens*, W *Bi* = Wild *B. impatiens*, *Br* = *B. rufocinctus, Bt* = *B. ternarius,* and *Bv* = *B. vagans*. Hosts with different letters are significantly different based on pairwise comparisons of estimated marginal means. (**C**) Principal coordinates analysis of phylotype community structure in bee gut microbiotas using Bray-Curtis dissimilarities. Bee hosts in the legend with different letters have significantly different community structures based on pairwise adonis analysis. For each plot, *n* = 39 individual bees and microbiotas; *n* = 4–12 per host.

We identified 10 phylotypes across all bee gut microbiotas: six core bumble bee gut phylotypes (*Bifidobacterium*, *Bombilactobacillus*, *Gilliamella*, *Lactobacillus*, *Schmidhempelia*, and *Snodgrassella*) and four non-core phylotypes (*Apibacter, Arsenophonus, Fructobacillus,* and *Lactobacillaceae CALYQJ01*) (Fig. 2A). The non-core phylotypes we observed are still known constituents of social bee gut microbiotas (11; NCBI GCA_945273735.1), though it is worth noting that this is only the second recorded observation of *Arsenophonus* spp. in bumble bee guts and the first of *Arsenophonus apicola* (44). In four of the 13 gut microbiotas containing *Arsenophonus*, this taxon dominated the microbial community, and these *Arsenophonus*-dominated microbiotas had higher 16S rRNA gene counts (average gene count in *Arsenophonus*-dominated *B. impatiens* and *B. rufocinctus* microbiotas: 9.9 × 10^8^; average gene count in all other *B. impatiens* and *B. rufocinctus* microbiotas: 7.4 × 10^7^).

This study is the first to characterize gut microbiotas of *B. rufocinctus, B. ternarius,* and *B. vagans* (Fig. 2A). Core phylotype constituents of these species' microbiotas included *Bifidobacterium* and *Gilliamella* in all three host species, *Schmidhempelia* in *B. vagans*, and *Apibacter* in *B. rufocinctus* and *B. vagans. Lactobacillus* was only observed in one *B. rufocinctus* individual and *Bombilactobacillus* was not observed in any species. *Snodgrassella* was also largely absent.

For *B. impatiens* hosts, major core phylotype constituents were *Bifidobacterium*, *Lactobacillus*, and *Schmidhempelia* (Fig. 2A)*. Bombilactobacillus* was absent from wild *B. impatiens* but present in commercial colonies, while *Snodgrassella* was absent from all Biobest bees but present in all Koppert and most wild *B. impatiens* individuals. *Bombilactobacillus* and *Gilliamella* were also missing in some individual Biobest and Koppert colonies.

Across all bee gut microbiotas sampled in this study, phylotype richness, but not Shannon diversity, varied with bee host (richness: likelihood ratio χ^2^ = 20.6, df = 5, *P* < 0.001; Shannon diversity: *F*_5,33_ = 1.57, *P* = 0.19) (Fig. 2B); neither metric varied with site for wild bee gut microbiotas (richness: likelihood ratio χ^2^ = 3.95, df = 5, *P* = 0.56; Shannon diversity: *F*_5,18_ = 0.75, *P* = 0.54). Specifically, wild *B. impatiens* gut microbiotas had higher phylotype richness than those of *B. rufocinctus* and *B. ternarius* (all |Z| > 3.00, all *P* < 0.05). Phylotype-level community structure varied across bee hosts (*F*_5,33_ = 4.82, *P* < 0.001, R^2^ = 0.42) (Fig. 2), with all hosts having significantly different community compositions from one another (all *F* > 2.49, all *P* < 0.05), except that *B. ternarius* community structure did not differ significantly from that of *B. rufocinctus* or wild *B. impatiens* (both *F* < 2.48, *P* > 0.08). The community structure of wild bee gut microbiotas did not vary with site (*F*_5,18_ = 1.12, *P* = 0.32), and community dispersions were similar across sites and hosts (all *F* < 1.1, all *P* > 0.3). Linear models revealed that the relative abundances of all phylotypes except *Bifidobacterium* varied with bee host (all *F*_5,33_ > 4.50, all *P* < 0.01), though post hoc analyses also showed no significant pairwise differences between bee hosts for the *Lactobacillaceae CALYQJ01* phylotype (Fig. 4; S3).

**Fig 3:**
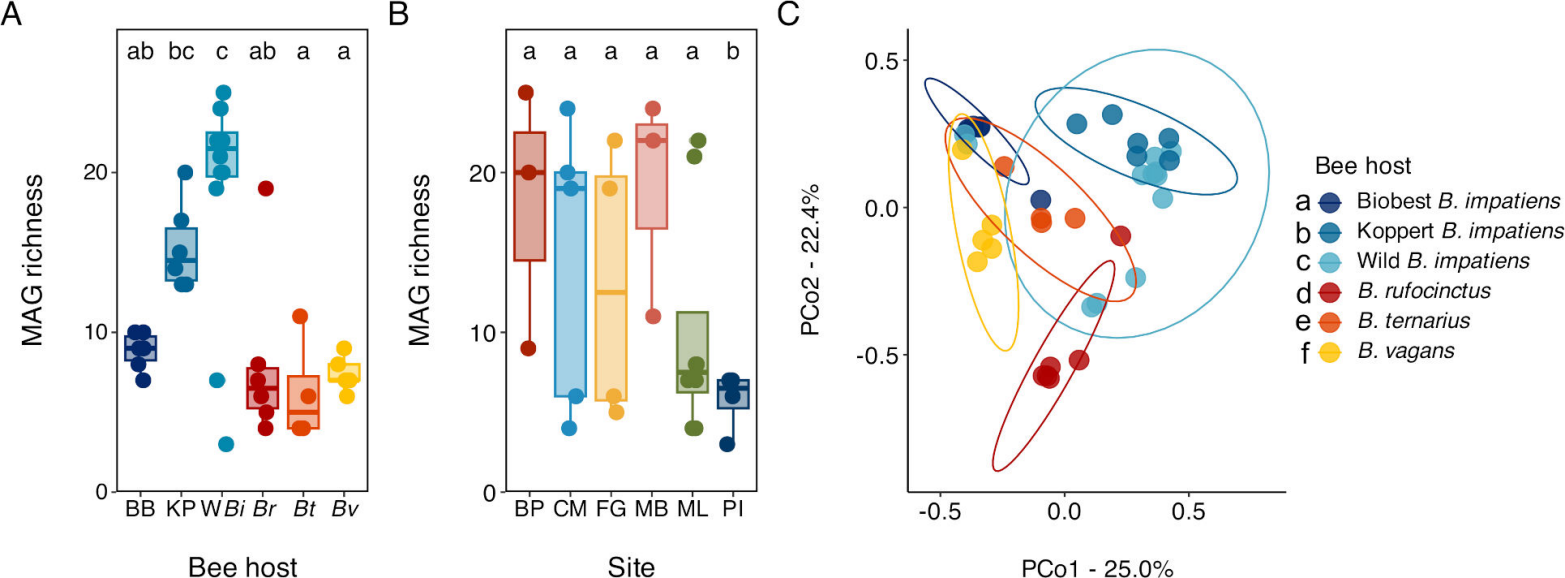
MAG richness and community structure in *Bombus* gut microbiotas. (**A**) Box plot of MAG richness by bee host in all bee gut microbiotas (*n* = 39; *n* = 4–12 per bee host). BB = BioBest *B. impatiens*, KP = Koppert *B. impatiens*, W *Bi* = Wild *B. impatiens*, *Br* = *B. rufocinctus, Bt* = *B. ternarius,* and *Bv* = *B. vagans*. (**B**) Box plot of MAG richness by site in wild bee gut microbiotas (*n* = 27; *n* = 3–8 per site). BP = Bruce Pit, CM = Chapman Mills Conservation Area, FG = Fletcher Wildlife Garden, MB = Mer Bleue Bog, ML = Mud Lake, PI = Petrie Island. For both box plots, boxes represent medians and interquartile ranges; whiskers extend to 1.5 × the interquartile range. Within each box plot, bee hosts and sites with different letters are significantly different based on pairwise comparisons of estimated marginal means. (**C**) Principal coordinates analysis of MAG community structure in all bee gut microbiotas using Bray-Curtis dissimilarities (*n* = 39; *n* = 4–12 per bee host). Bee hosts in the legend with different letters have significantly different community structures based on pairwise adonis analysis.

The 10 phylotypes we identified comprised 32 representative MAGs; the number of MAGs in each phylotype ranged from 1 to 7 (Fig. S4). MAG richness varied with bee host (likelihood ratio χ^2^ = 54.2, df = 5, *P* < 0.0001) and, in wild bees, with the site (likelihood ratio χ^2^ = 31.1, df = 5, *P* < 0.0001) (Fig. 3). Specifically, for all bee hosts, wild *B. impatiens* gut microbiotas had higher MAG richness than all other hosts except Koppert *B. impatiens* (all |Z| > 4.10, all *P* < 0.001), and Koppert *B.* impatiens gut microbiotas had higher MAG richness than those of *B. ternarius* and *B. vagans* (all |Z| > 3.10, all *P* < 0.05) (Fig. 3A). For wild bee hosts only, MAG richness was lower at Petrie Island than any other site (all |Z| > 3.30, all *P* < 0.01) (Fig. 3B). MAG Shannon diversity did not vary significantly with bee host or site (host: *F*_5,33_ = 2.39, *P* = 0.06; site: *F*_5,18_ = 1.00, *P* = 0.45).

**Fig 4:**
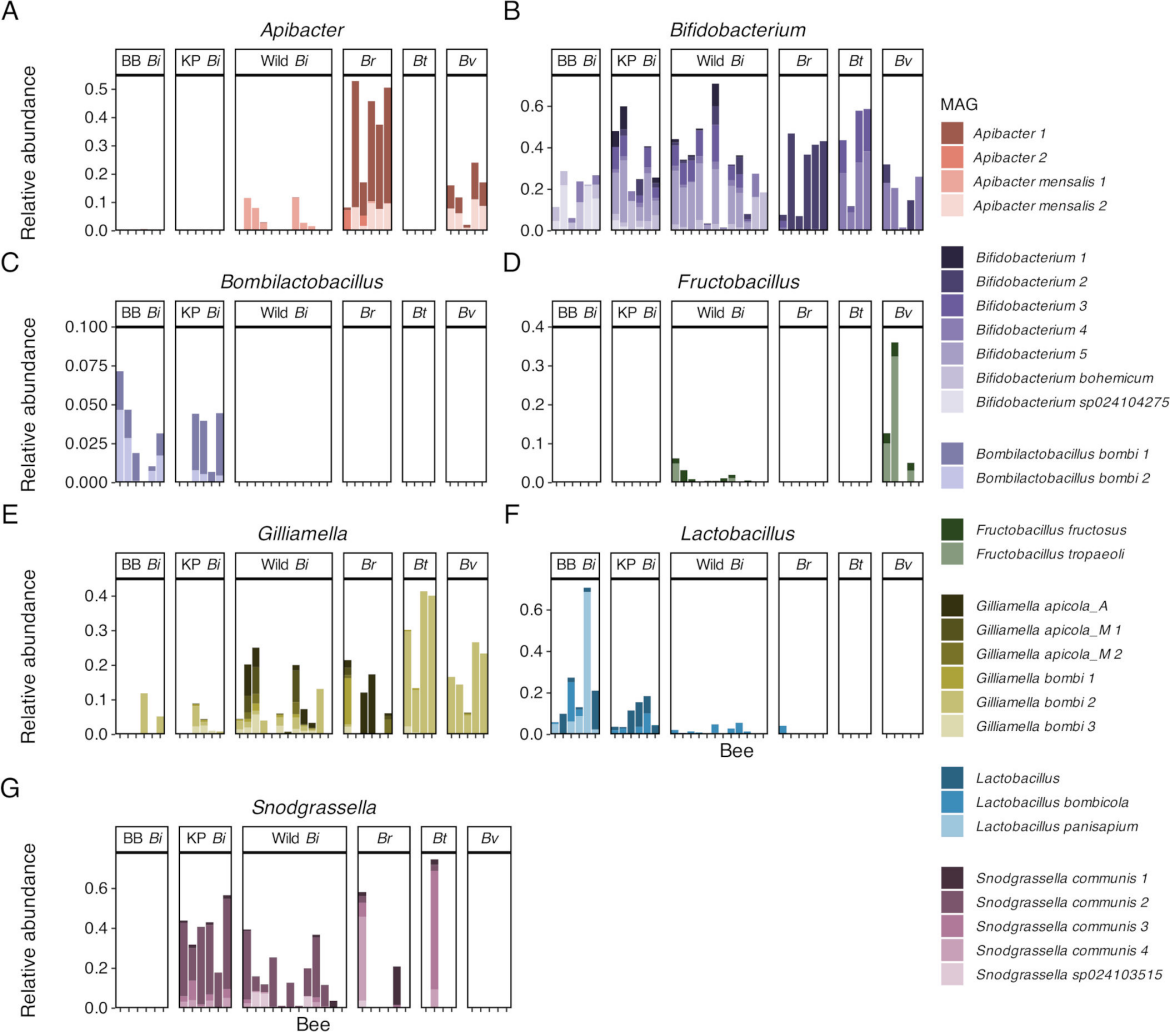
Stacked bar plots of relative abundances of representative MAGs in *Bombus* gut microbiotas for phylotypes with more than one representative MAG. BB *Bi* = Biobest *B. impatiens*, KP *Bi* = Koppert *B. impatiens*, Wild *Bi* = Wild *B. impatiens*, *Br* = *B. rufocinctus, Bt* = *B. ternarius,* and *Bv* = *B. vagans*. As *Arsenophonus*, *Lactobacillaceae CALYQJ01*, and *Schmidhempelia* were all represented by only one MAG, the variation in MAG relative abundance is the same as the variation in phylotype relative abundance (see Fig. S5-5).

MAG-level community structure varied across bee hosts (*F*_5,33_ = 6.94, *P* < 0.001, R^2^ = 0.51) (Fig. 3C; 4) but did not vary with site in wild bee gut microbiotas (*F*_5,18_ = 1.24, *P* = 0.21); group dispersions did not vary for either variable (all *F* < 1.5, all *P* > 0.2). Specifically, all hosts had significantly different community compositions from one another (all *F* > 2.8, all *P* < 0.05). Though not statistically analyzed, visually the relative abundance of all representative MAGs varied strongly with bee host, with all MAGs being completely absent from at least one bee host (Fig. 4). This pattern held true even for MAGs of *Bifidobacterium*, which was consistently present across hosts at the phylotype level (Fig. S3C).

### Metabolic potential of MAG clusters

We had 103 high-quality MAGs (i.e., >80% completeness and <10% contamination) comprising 32 MAG clusters, with the number of MAGs in each MAG cluster ranging from 1 to 14. Half of these MAGs (52) were generated from wild *B. impatiens* gut microbiotas, 18 from Koppert *B. impatiens*, 13 from Biobest *B. impatiens*, 13 from *B. rufocinctus*, 5 from *B. ternarius,* and 2 from *B. vagans.* Across all MAG clusters, we identified 104 KEGG modules belonging to eight module categories. The presence of these KEGG modules varied significantly by MAG cluster (*F*_31,71_ = 31.4, *P* < 0.001, R^2^ = 0.93); MAG cluster dispersions did not vary (*F*_31,71_ = 1.14, *P* = 0.33). Enrichment analysis revealed that of the 104 identified modules, 100 were differentially enriched across MAG clusters (all *P* < 0.05). Almost all modules were enriched in MAG clusters belonging to at least two different phylotypes, showing a high level of functional redundancy across phylotypes. Of the non-differentially enriched modules, two had consistently low prevalence (heme biosynthesis in animals and fungi, and NAD biosynthesis beginning with tryptophan, both modules within cofactor metabolism) and two were consistently present (PRPP biosynthesis, a carbohydrate metabolism module, and adenine ribonucleotide biosynthesis, a nucleotide metabolism module).

### Metabolic potential of bee gut microbiotas

**Fig 5:**
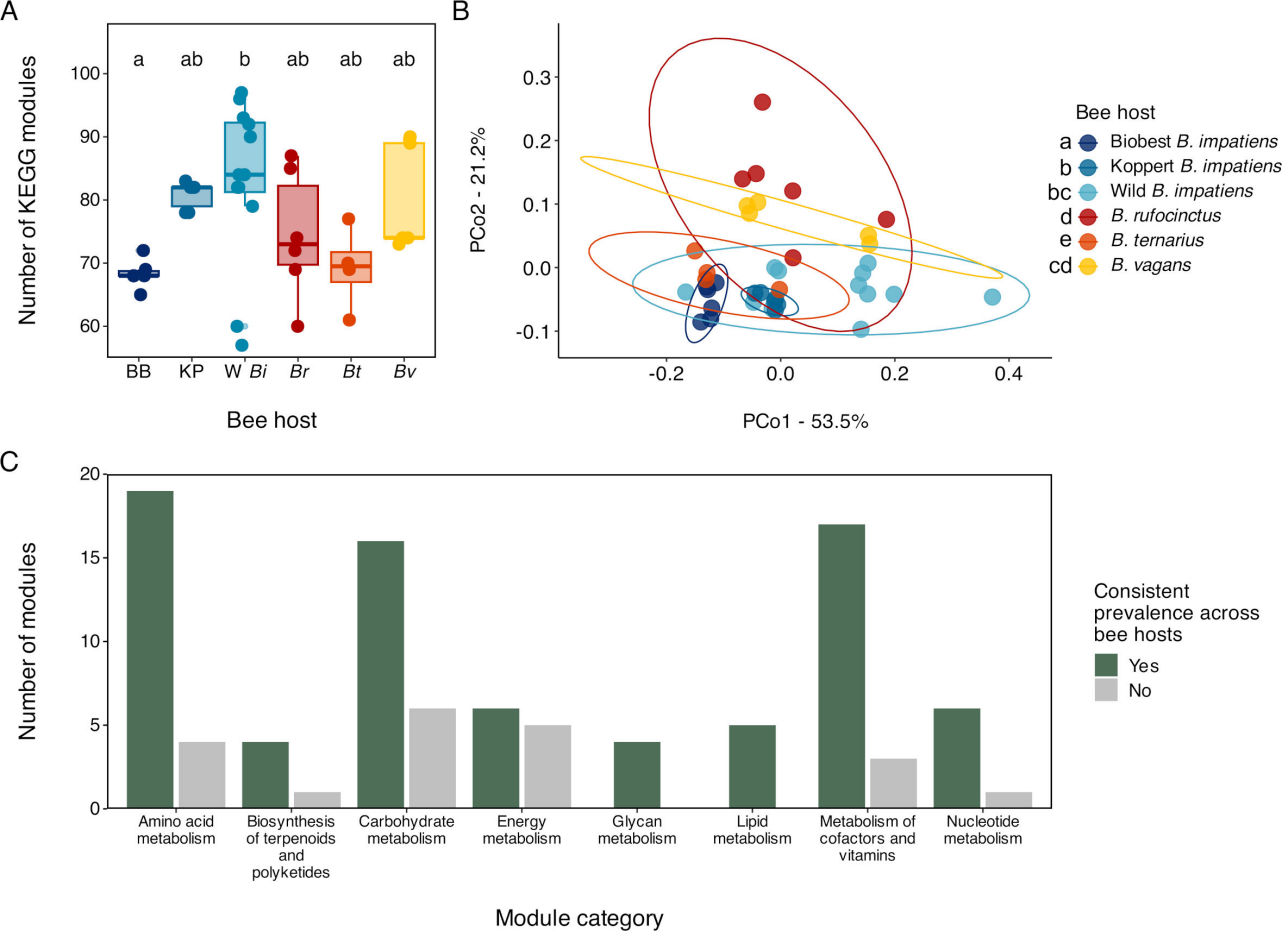
Whole community metabolism of bumble bee gut microbiotas. (**A**) Box plot of the number of KEGG modules expected to be present in bee gut microbiotas by bee host (*n* = 39; *n* = 4–12 per bee host). BB = Biobest *B. impatiens*, KP = Koppert *B. impatiens*, W *Bi* = Wild *B. impatiens*, *Br* = *B. rufocinctus, Bt* = *B. ternarius,* and *Bv* = *B. vagans*. Boxes represent medians and interquartile ranges; whiskers extend to 1.5 × the interquartile range. Bee hosts with different letters are significantly different based on pairwise comparisons of estimated marginal means. (**B**) Principal coordinates analysis of KEGG module presence/absence in all bee gut microbiotas using Jaccard distances (*n* = 39; *n* = 4–12 per bee host). Points are jittered for visibility. Bee hosts with different letters in the legend have significantly different community structures based on pairwise adonis analysis. (**C**) Bar plot of the number of KEGG modules in all bee gut microbiotas that are and are not differentially enriched in bee hosts.

We identified 97 KEGG modules that had at least a 75% probability of being present in at least one bee. The number of modules expected to be present in each bee gut microbiota ranged from 57 to 99 and varied with bee host (likelihood ratio χ^2^ = 16.9, df = 5, *P* = 0.005) (Fig. 5A). Specifically, wild *B. impatiens* gut microbiotas contained higher numbers of unique KEGG modules than Biobest *B. impatiens* gut microbiotas (|Z| = 3.31, *P* = 0.01). In addition, bee gut microbiota KEGG module composition varied with bee host (*F*_5,33_ = 6.02, *P* < 0.001, R^2^ = 0.48) (Fig. 5B), though not to the same extent as phylotype or MAG-level taxonomic community structure. Neither the number of KEGG modules nor KEGG module composition varied by the site for wild bee gut microbiotas (number: likelihood ratio χ^2^ = 1.52, df = 5, *P* = 0.91; composition: *F*_5,18_ = 1.54, *P* = 0.13).

Enrichment analysis revealed that of the 97 KEGG modules present across bee hosts, only 20 (21%) were differentially enriched (all adjusted q-values < 0.05); the other 77 had similar prevalence across all bee hosts (Fig. 5C). Of those 77 modules, 61 were consistently present (Fig. S5) and 16 had consistently low prevalence (all adjusted q-values > 0.05). Of the 61 modules that were consistently present, 44 (72%) were present in every individual in every host. All differentially enriched modules were enriched in at least two bee hosts (Fig. S6). Koppert and wild *B. impatiens* gut microbiotas contained the greatest number of enriched modules, 17 each; *B. vagans* had 11, *B. rufocinctus* had nine, *B. ternarius* had seven, and Biobest *B. impatiens* had six.

## DISCUSSION

### Gut microbiota community structure varies with species in wild bumble bees

We found that gut microbial community composition and structure varied between wild bumble bee species sampled from the same geographic area. At phylotype resolution, composition and community structure were relatively consistent within, but clearly differed between, species (Fig. 2A and C). Many of these phylotypes were composed of multiple sequence-discrete populations (i.e., representative MAGs) whose prevalence and abundance also varied with species (Fig. 4); thus, gut microbiotas were even more distinct between species at the MAG level (Fig. 3C). Intra-phylotype variation is often not fully captured using standard 16S rRNA gene amplicon sequencing (74, 75), and our results highlight the loss of information that results from examining gut microbiotas at coarser taxonomic resolutions. Overall, our results suggest that the wild bumble bee species surveyed in our study have species-specific core gut microbiotas composed of unique combinations of bee-associated microbial phylotypes and phylotype populations, as seen in other bumble bee communities (38, 66).

Profiled for the first time in this study, *B. rufocinctus*, *B. ternarius*, and *B. vagans* gut microbiotas were quite different from those typically observed in bumble bees (11, 12, 38). Notably, most *B. rufocinctus* and *B. ternarius* individuals and all *B. vagans* individuals lacked *Snodgrassella*, a striking result given this phylotype is highly prevalent in most if not all other profiled bumble bee species (11, 12, 37, 38, 66). *Snodgrassella* has been isolated from *B. vagans* previously (62), so its absence from this species in our study may be population-specific; more extensive sampling would provide clarification. Another previous study (52) documented an absence of *Snodgrassella* in bumble bees that had recently eclosed (i.e., emerged from pupation). However, newly eclosed workers do not forage until a few days post-eclosion (119), and we surveyed foraging bees during peak colony activity when older workers were abundant. Thus, it is unlikely that young worker age explains the lack of *Snodgrassella* in our study. *Lactobacillaceae* phylotypes were also rare in all three species (Fig. 2), though this is a more common observation, especially for *Bombilactobacillus* (12, 38). In fact, a lack of *Bombilactobacillus* in these species lends support to a hypothesis that *Bombilactobacillus* was lost in a common ancestor of many “short-faced” bumble bees (12, 85). Meanwhile, two *Apibacter* MAGs were present in all *B. vagans* and *B. rufocinctus* individuals, and at higher relative abundances than is typically seen in most bumble bee gut microbiotas (12, 40). *Apibacter* is a genus of corbiculate-bee-associated microbes whose presence has been associated with decreased pathogen infection in *B. impatiens* (41, 43, 47, 120). While currently considered a rare, non-core member of bumble bee microbiotas (47), the prevalence of *Apibacter* in *B. rufocinctus*, *B. vagans*, and the recently surveyed *B. friseanus* (66) suggests this phylotype may constitute part of the core gut microbiota for some bumble bee species or populations, joining the likes of *Schmidhempelia* and *Bombiscardovia*. However, we sampled a small number of individuals from these species over a limited geographic area and time period; more extensive sampling would be required to support this hypothesis.

Another interesting phenomenon we observed was the presence of *Arsenophonus apicola*-dominated gut microbiotas in *B. impatiens* and *B. rufocinctus* (Fig. 2A). While other *Arsenophonus* species have been found in bumble bee guts (121), this is the first recorded instance of *Arsenophonus apicola* (122). The *Arsenophonus* genus contains symbiotic bacteria primarily associated with insects, and their relationships with their hosts range from parasitism to mutualism (123). In bees, *Arsenophonus* is considered an environmental phylotype found in flowers (124), solitary bees (125), and social bee midguts (12, 126) that shows few signs of vertical transmission in bee hosts (126). Thus, it appears that *Arsenophonus*-dominated microbiotas represent a new variation of a recurring gut enterotype in bumble bees wherein environmental microbes constitute most of the gut microbiota (35, 38)—a shift that may adversely affect host health (127, 128). However, typically in these environmental enterotypes, core phylotypes are mostly or completely displaced (35, 38). We found that core gut phylotypes were still present in *Arsenophonus*-dominated gut microbiotas, while overall microbial abundances were an order of magnitude higher than in non-*Arsenophonus*-dominated microbiotas within the same host species. These observations imply that a large *Arsenophonus* population is present in addition to, not instead of, core phylotypes, perhaps growing in a gut compartment other than the core-dominated hindgut (126). Further research is needed to determine where *Arsenophonus* resides in the bumble bee gut and the effect, if any, of such a large population on host health and performance.

### Commercial and wild *B. impatiens* have different gut microbial communities

Gut microbiota community structure differed not only among species but also with bee origin (i.e., commercial or wild) within the same species, and between commercial suppliers. Consistent with previous studies, the gut microbiotas of wild *B. impatiens* were generally dominated by *Bifidobacterium*, *Gilliamella*, *Lactobacillus*, *Schmidhempelia*, and *Snodgrassella*, with environmental bacteria as minor constituents (14, 40). As expected, commercial colonies did not contain any non-core or environmental phylotypes (Fig. 2A). Instead, all but one commercial colony contained *Bombilactobacillus*, a phylotype that is often present in commercial colonies and absent in wild *B. impatiens* (40, 43, 49). Beyond phylotype differences, some *Bifidobacterium*, *Gilliamella*, and *Lactobacillus* MAGs were restricted to either commercial or wild *B. impatiens*, and, in some cases, were largely restricted to specific suppliers, as with *Bifidobacterium sp024104275* and *Lactobacillus apium* in Biobest bees.

However, the most striking difference in phylotype composition was the complete lack of *Snodgrassella* in all Biobest bees (Fig. 2A). *Snodgrassella* is typically prevalent at high relative abundances in *B. impatiens* gut microbiotas (40), including those sampled from Biobest colonies (48, 49). While an absence of *Snodgrassella* has been observed once before in a Biobest colony (49), it has never been observed in multiple colonies purchased together. Still, we believe this result, while unusual, to be real: all colonies were well established so it is unlikely all six individuals were newly-eclosed workers with still-assembling gut microbiotas, and no high-quality *Snodgrassella* MAGs were generated from Biobest sequence data.

We also found that individual commercial colonies could lack additional core phylotypes. One colony from each supplier did not contain *Gilliamella*, and even in the colonies where it was present, within-phylotype diversity was much lower than in wild *B. impatiens* (Fig. 2A and 4). In some bumble bee colonies, *Gilliamella* can take up to 2 weeks to establish in bee guts after eclosion (52); therefore, these absences could be due to age effects. However, *Gilliamella* can also be established much earlier (73), and a consistent lack of *Gilliamella* over multiple weeks has been seen in other colonies (49). While noted before in Biobest colonies (48, 49), this is the first observed *Gilliamella* absence in Koppert colonies. The same Koppert colony missing *Gilliamella* was also missing *Bombilactobacillus*, a phylotype that was found in all other commercial bees.

The complete absence of common core phylotypes in some commercial colonies is noteworthy. Core social bee gut phylotypes vary in their metabolisms (24, 26, 28) and sensitivity to external stimuli (49, 129–131). As such, the presence of certain phylotypes is important for investigating certain topics, such as the presence of *Snodgrassella* for investigating the impacts of glyphosate exposure (48, 80, 132, 133). The fact that multiple commercial colonies could be ordered for an experiment and some, possibly all, could lack a crucial phylotype is troubling. The absence of core phylotypes could also alter microbial community dynamics, limiting the relevance of certain studies in commercial *B. impatiens* colonies to wild *B. impatiens* populations. To mitigate this risk, we recommend that, if possible, researchers purchase commercial colonies for experiments in multiple batches at different time points, and, if a specific phylotype is required for an experiment, its presence be confirmed using culturing or molecular techniques prior to experimentation. In addition, we recommend that future research be dedicated to identifying factors driving core phylotype loss in commercial bees. Core phylotypes can decline dramatically in abundance and even be lost completely in diapausing queen bumble bees (134–136), who seed the gut microbiotas of their workers (73); thus, diapause may be a good place to begin these investigations.

Overall, although wild *B. impatiens* and bees from the two commercial suppliers all harbored distinct gut microbiotas, gut microbiotas from Koppert colony bees were the most like those of wild *B. impatiens* (Fig. 2B and 3C). However, we sampled a small number of workers from only a few colonies purchased at one time, and some of the differences that made Biobest bee gut microbiotas so distinct in this study (i.e., the lack of *Snodgrassella*) are rare. More extensive sampling of commercial colonies purchased at multiple time points is required to verify and clarify the patterns we observed.

### Metabolic potential is largely consistent across bee hosts

We found that taxonomic differences in bumble bee gut microbiotas between bee hosts did not correspond to differences in metabolic potential. Despite significant differences in gut microbiota taxonomic richness and community structure between bee hosts, almost 80% (77/97) of all metabolic modules identified in bee gut microbiotas had either consistently high or low prevalence across all hosts (Fig. 5C; Fig. S5). In fact, of the 61 modules that were consistently present, more than two-thirds were present in all surveyed individuals (Fig. S5), suggesting a high degree of stability in metabolic function both within and across hosts. Our observations match those from a wide variety of environmental and host-associated microbiotas in which community function remains stable within environments despite fluctuations in taxonomic composition and structure (73, 137–139); this stability is due to metabolic redundancy across microbial taxa, which we also observed in our phylotypes and MAG clusters.

The variable prevalence of core phylotypes but largely consistent metabolic potential supports the idea of a flexible bumble bee core gut microbiota in which multiple combinations of phylotypes can create communities capable of similar functions and support of host health. In fact, in bumble bees, gut microbial contributions to host performance are primarily associated with fundamental microbial metabolisms, such as organic acid and glycerophospholipid biosynthesis (15, 31). Modules that produce important precursors in these biosynthetic pathways, such as glycolysis, pyruvate oxidation, and phosphatidylethanolamine biosynthesis, were—unsurprisingly—consistently present across all hosts (Fig. S5). Consistent metabolic potential across bumble bee hosts also implies that for certain types of research, such as fundamental work describing the effects on bumble bee function and performance of having or lacking a gut microbiota, research conducted on one host population is likely relevant to many other bumble bee populations. However, relevance could still be limited by phylotype-specific responses to experimental conditions as discussed previously.

Though metabolic potential was largely consistent across bee hosts, there were metabolic modules that were differentially enriched (Fig. 5C). Some of these modules, like those for isoleucine, lysine, C5 isoprenoid, and pyrimidine biosynthesis, were functionally redundant with other non-differentially enriched modules; the same metabolic process could be achieved through multiple pathways (Fig. S5 and S6). Thus, their differential presence may have little effect on community function. However, the differential enrichment of other modules may have implications for gut microbiota and/or host performance. For example, microbiotas that can synthesize their own cysteine, a key amino acid, or pantothenate, an essential B-vitamin, may better tolerate environmental fluctuations in the availability of these organic molecules, as may their hosts (140–145). This potential variation in performance may even be linked to well-documented variations in environmental niches occupied by different bee hosts (e.g., 10, 146). Still, these effects and links remain purely speculative; further studies are required to determine whether and to what degree the presence or absence of these differentially enriched modules affects gut microbiota performance and bee host health.

Our study presents an initial look at metabolic potential across bumble bee gut microbiotas, but there are limitations to our methodology. The KEGG module database that we used to compare metabolic potential is composed of a limited number of genes and gene sets. Thus, a wide variety of enzymes and metabolic pathways were excluded from our analysis. Further investigation of metabolic potential using other gene databases will provide a more comprehensive understanding of how the community-level functional potential of bumble bee gut microbiotas changes with bee hosts (101, 147, 148). However, even with more extensive gene and pathway annotation, all discussions of microbiota metabolism would remain speculative. First, the genomes we analyzed were metagenome-assembled genomes and may not represent real genomes of microbes from our sampled communities (149). Furthermore, we analyzed changes in gene presence and absence, not gene activity, so we do not know if the genes we observed were expressed. Future experiments should employ metatranscriptomics, metabolomics, and metaproteomics to determine whether the consistency we observed in metabolic potential corresponds to consistency in gene expression and community function.

### Conclusion

We found that gut microbiota community composition and structure varied with bumble bee species and origin (i.e., commercial vs. wild), and between commercial suppliers. However, despite these differences, metabolic potential was largely consistent across bee hosts. Our observations support the idea of a flexible bumble bee core gut microbiota in which multiple combinations of core phylotypes can create communities that function, and thereby support host health and performance, in similar ways.

Altogether, our results provide new insights into the structure and function of bumble bee gut microbiotas. Bumble bees are key pollinators, and their gut microbiotas are important components of their health and performance. As we continue to research this symbiosis, the results presented here will be useful for evaluating how knowledge gained from studying one bumble bee population may translate to other host populations and species.

## Data Availability

Supplemental tables, figures, and captions are available at doi.org/10.6084/m9.figshare.c.7484784.v1. All data, R scripts, and MAGs are available at https://github.com/michellehotch/AEM-2024. Raw sequences are available through the NCBI Sequence Read Archive (PRJNA1044797).
